# Network for Therapy in Rare Epilepsies (NETRE): Lessons From the Past 15 Years

**DOI:** 10.3389/fneur.2020.622510

**Published:** 2021-01-14

**Authors:** Celina von Stülpnagel, Andreas van Baalen, Ingo Borggraefe, Kirsten Eschermann, Till Hartlieb, Lorenz Kiwull, Milka Pringsheim, Markus Wolff, Manfred Kudernatsch, Gert Wiegand, Pasquale Striano, Gerhard Kluger, NETRE Consortium

**Affiliations:** ^1^Division of Pediatric Neurology, Developmental Medicine and Social Pediatrics, Department of Pediatrics and Epilepsy Center, Dr. von Hauner Children's Hospital, Ludwig-Maximilians-University, Munich, Germany; ^2^Institute for Transition, Rehabilitation and Palliation, Paracelsus Medical University, Salzburg, Austria; ^3^Clinic for Child and Adolescent Medicine II, University Hospital Schleswig-Holstein, Kiel, Germany; ^4^Center for Pediatric Neurology, Neurorehabilitation and Epileptology, Schoen Klinik Vogtareuth, Vogtareuth, Germany; ^5^Institute of Social Pediatrics and Adolescent Medicine, Ludwig-Maximilian-University, Munich, Germany; ^6^Department of Pediatric Neurology, Vivantes Hospital Neukölln, Berlin, Germany; ^7^Clinic for Neurosurgery, Schön Klinik Vogtareuth, Vogtareuth, Germany; ^8^Neuropediatrics Section of the Department of Pediatrics, Asklepios Clinic Hamburg Nord-Heidberg, Hamburg, Germany; ^9^Pediatric Neurology and Muscular Diseases Unit, Istituto die Ricovero e Cura a Carattere Scientifico Istituto Giannina Gaslini, Genova, Italy; ^10^Department of Neurosciences, Rehabilitation, Ophthalmology, Genetics, Maternal and Child Health, University of Genova, Genova, Italy

**Keywords:** NETRE, FIRES (Febrile infection epilepsy-related syndrome), SYNGAP1, SYN1, FOXG1, SCN2A, PATRE, Early Neuroimpaired Twin Entity (ENITE)

## Abstract

**Background:** In 2005, **Ne**twork for **T**herapy in **R**are **E**pilepsies (NETRE)—was initiated in order to share treatment experiences among clinicians in patients with rare epilepsies. Here we describe the structure of the rapidly growing NETRE and summarize some of the findings of the last 15 years.

**Methodology/Structure of NETRE:** NETRE is organized in distinct groups (currently >270). Starting point is always a patient with a rare epilepsy/ epileptic disorder. This creates a new group, and next, a medical coordinator is appointed. The exchange of experiences is established using a data entry form, which the coordinator sends to colleagues. The primary aim is to exchange experiences (retrospectively, anonymously, MRI results also non-anonymously) of the epilepsy treatment as well as on clinical presentation and comorbidities NETRE is neither financed nor sponsored.

**Results:** Some of the relevant results: (1) first description of FIRES as a new epilepsy syndrome and its further investigation, (2) in *SCN2A*, the assignment to gain- vs. loss-of-function mutations has a major impact on clinical decisions to use or avoid treatment with sodium channel blockers, (3) the important aspect of avoiding overtreatment in *CDKL5* patients, due to loss of effects of anticonvulsants after 12 months, (4) pathognomonic MRI findings in *FOXG1* patients, (5) the first description of pathognomonic chewing-induced seizures in *SYNGAP1* patients, and the therapeutic effect of statins as anticonvulsant in these patients, (6) the phenomenon of another reflex epilepsy—bathing epilepsy associated with a *SYN1* mutation. Of special interest is also a NETRE group following twins with genetic and/or structural epilepsies [including vanishing-twin-syndrome and twin-twin-transfusion syndrome) [= “Early Neuroimpaired Twin Entity” (ENITE)].

**Discussion and Perspective:** NETRE enables clinicians to quickly exchange information on therapeutic experiences in rare diseases with colleagues at an international level. For both parents and clinicians/scientist this international exchange is both reassuring and helpful. In collaboration with other groups, personalized therapeutic approaches are sought, but the present limitations of currently available therapies are also highlighted. Presently, the PATRE Project (PATient based phenotyping and evaluation of therapy for Rare Epilepsies) is commencing, in which information on therapies will be obtained directly from patients and their caregivers.

## Introduction

The definition of *rare* or *orphan disease* varies among international public health systems, with an estimated prevalence of affected patients between 1:1,000,000 and 1:200,000 persons. In Europe, the number determined is <5 patients per 10,000 people. Summing up every rare disease ultimately results in a count that appears as *not so r*are. Just in Germany, for instance, there are about 7,000 different rare diseases, affecting four million patients (1). Several hundred of these rare diseases are associated with epileptic seizures and epileptic encephalopathies in infancy and childhood. New diagnostic tools, such as Next Generation Sequencing (NGS) and exome sequencing, have increasingly enabled the identification of new genes for various rare diseases. Currently, about 30–40% of epilepsy syndromes have an associated genetic mutation, and, in fact, the number of new genetic findings is increasing enormously (2). Thus, treating physicians are often faced with only recently discovered syndromes, while lacking information about optional treatment options. Furthermore, high throughput genetic testing might result in the finding of variants of unclear significance (3).

In 2005, a network among pediatric epileptologists—**Ne**twork for **T**herapy in **R**are **E**pilepsies (NETRE; www.netre.de)—was initiated by author GK with the primary aim of exchanging treatment experiences among clinicians in patients with very rare epilepsies. We started with three patients whose epilepsies we later named “Febrile Infection-Related Epilepsy Syndrome” (FIRES) (4, 5). Since then, the primary goal has remained the exchange of treatment experience among clinicians for patients with very rare genetic epilepsies, and the collection of data on genetic findings, clinical presentation, and comorbidities. Here we describe the structure of the growing NETRE and summarize the experiences of the last 15 years as case examples.

## Methodology and Structure of NETRE

**Figure 1:**
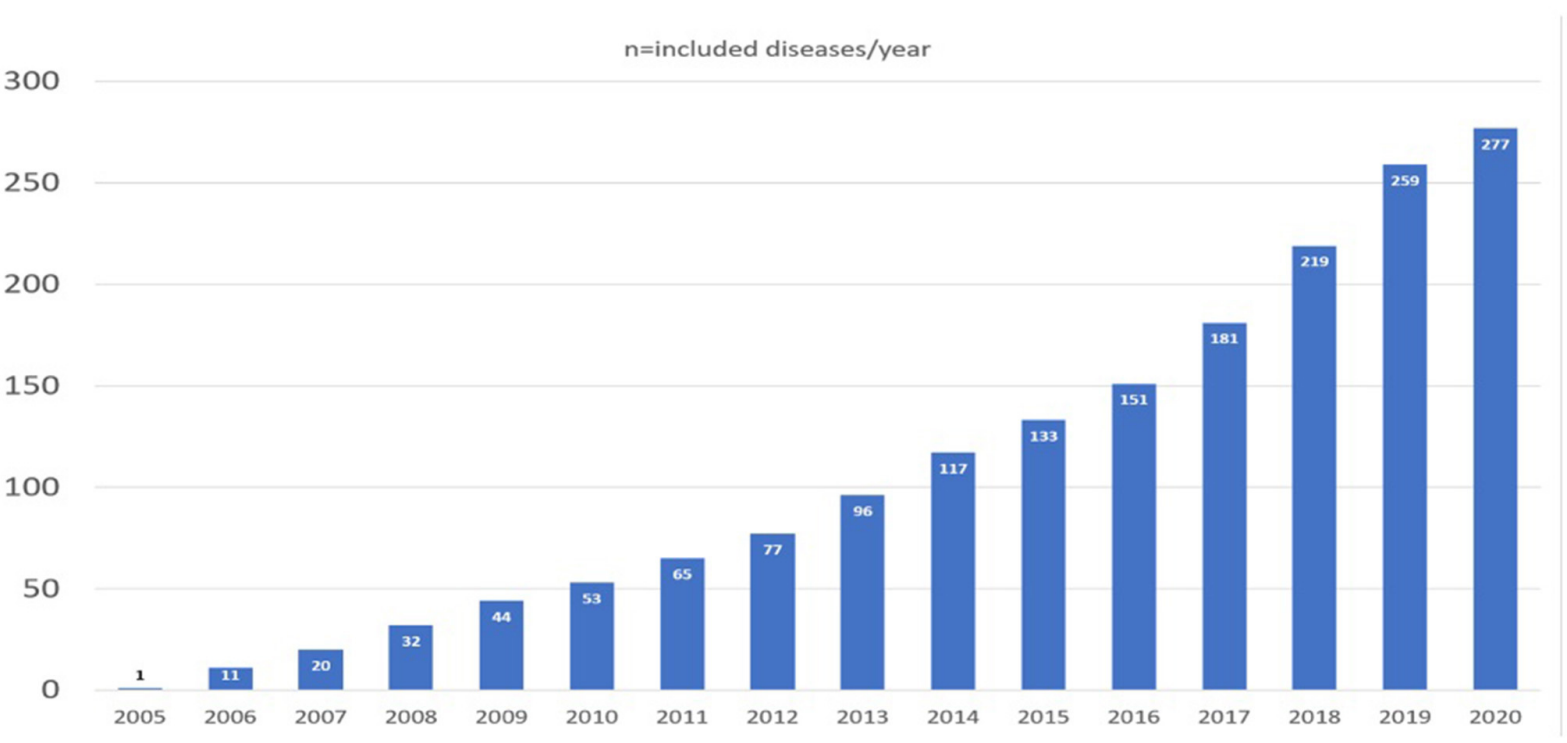
Overview of the increase in the different NETRE-groups over time. In 2020, only the first 6 months were included.

**Figure 2:**
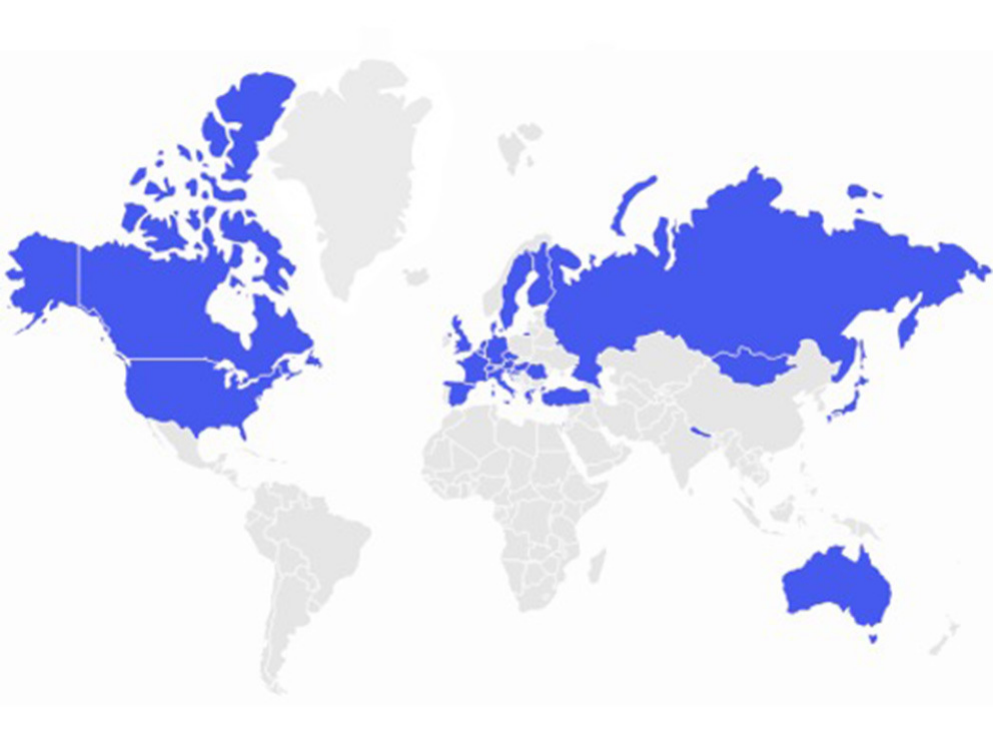
NETRE- map of the countries of origin of participating colleagues.

The organizational structure of NETRE is made up of groups, which are led by a medical coordinator. The starting point is always a patient with a rare epilepsy or epileptic disorder. This creates a new group and then a medical coordinator is appointed. The exchange of experiences is done using a data entry form, which the coordinator sends to colleagues that are treating a patient with the same rare epilepsy and communication is done via email. To find a list of the current disorders there is a homepage (www.netre.de) or information is provided by direct contacting GK via email. The primary aim is to exchange experiences on the treatment of epilepsy in these rare diseases based on anonymized patient's data. This procedure has been approved by the Ethics Committee of the Bavarian Medical Association. NETRE is neither financed nor sponsored. NETRE is currently investigating more than 338 “orphan diseases” (caused by single-gene mutations, numerical or structural chromosomal anomalies, metabolic disorders or syndromes that are still etiologically unclear) presenting with epileptic seizures (Table 1, Figure 1). In addition to the question of targeted anticonvulsant therapy (including ketogenic diet or epilepsy surgery), the individual groups are interested in various clinical and scientific issues (e.g., MRI findings, epilepsy characteristics, clinical findings), investigated by numerous collaborations with other research groups and self-help organizations (Figure 2). The findings within NETRE have been published in more than 40 papers (Table 2). Every 3 months the members of NETRE are informed of new projects or publications by a newsletter.

**Table 1:** Overview of active NETRE groups.

**Single Genmutation**	ADCY5; ADPRHL2; AGC1; AKT1; AKT3; ALG13; ALDH5A1; AMT; ANKRD11; ARHGEF9; ARID1A/ARID1B; ARV1; ARX; ASXC1; ATP1A2; ATP1A3; ATP6V1B2; ATRX; AUTS2; BRAT1; CACNA1A; CACNA1B; CACNA1D; CAD; CAMK4; CASK; CDKL5; CHD2; CHD7; CHRNA4; CHNRB2; CLN1-14; CNKSR2; CNTNAP2; COG7 (CDG2E); COL2A1; COL4A1; COL4A2; COQ4; CSTB; CUX2; CYFIP2; DCX; DDX3X; DEPDC5; DHDDS; DNM1; DNM1L; EFTUD2; EHMT1; FASTKD2; FBOX11; FLNA; FOLR1; FOXG1; FRRS1; GABRA1; GABRA4; GABRG2/3; GABRB3; GAD2; GCSH / GLDC; GFAP; GLRA1; GNAO1; GNB5; GRIN1; GRIN2A; GRIN2B; HNRNPU; HUWE1; IDIC15; IKBKG; IQSEC2; KAT6A; KCHN1; KCNA2; KCNB1; KCNC1; KCNC2; KCNJ10; KCNMA1; KCNQ1; KCNQ2 / Q3; KCNT1; KCTD7; KIAA2022; KIFSCM; KPTN; KRAS; KRIT1; LIS1; MBD5; MECP2; MEFC 2; MT-ND3: MT-ND6; MTOR; NALCN; NAPB; NBEA; NDUFS2; NEXMIF; NFIX; NIPA2; NPC1/2; NPRL2, 3; NRXN1; PACS1; PARP6; PCDH12; PCDH19; PDH; PDHA1; PIGA; PIGN; PIGT; PIGC; PIGG; PIGH; PIGP; PIGY; PGAP1; PIGV; PIK3R2; PLP1; PMKP; PNPO; POLG; POMGNT1; PPP2CA; PPP3CA; PROSC; PRRT2; PRUNE; PRUNE1; PURA; QARS; RAI1; RALGAPA1; RANBP2; RHOBTB2; RNASEH2A; RNASEH2B; RNASEH2C; RNF13; ROGDI; RORB; RPS6KA3; SAMHD1; SCA2; SCA7; SCN1A; SCN2A; SCN5A; SCN8A; SCN9A; SERAC1; SHANK3; SLC12A5; SLC13A5; SLC19A3; SLC1A2; SLC25A12; SLC25A22; SLC2A1; SLC35A2; SLC6A1; SLC7A7; SLC9A6; SMARCA2; SMARCA4; SMARCB1; SMARCE1; SMC1A; SPATA5; SPTAN1; STX1B; STXBP1; SYN1; SYNGAP1; SYT1; TANGO2; TBC1D24; TBCK;; TBR1; TCF4; TCTN1; TDCBD24; TREX1; TRIO; TTC19; TUB1A; TUBB2B; TUBB3; TUBB6; UBTF; UNC80; USP9X; WDR45; WWOX; YWHAG; ZBTB18; ZFHX1B, ZEB2; ZYMND11;
**Structural Chromosomal Anomalies**	1 p13.3p13.2 deletion; 1q21.3; 1q36; 1q44 deletion; 2q deletion; 2q22.3; 2q24 duplication; 4p-, 4p+: 5p duplication; 5p15.2 deletion; 5q14.3 deletion; 5q31.3 deletion; 7q35; 10q deletion; 12p13.2 deletion; tetrasomie 12 p(mosaic); 14q32 duplication; 15q11.1 deletion; 15q11.2 duplication; 15q13.3 deletion; Inversion 15, Iso or Markerchromosom 15; Monosomie 15q;16p11.2 deletion; 16p12.2 microdeletion; 16p13.1 deletion; 18q deletion; 19p13 duplication; 21 q deletion; 22q13.1 duplication; 22q13.3 deletion; Del Macro D2; Ringchromosome 14; Ringchromosome 20; Trisomie 8; Triple X, XYY.
**Syndromes**	Aicardi Syndrome; Aicardi- Goutières Syndrome; Alport Syndrome; Alternating hemiplegia; Angelman Syndrome; Bardet-Biedl Syndrome; Beckwith-Wiedemann-Syndrome; CAPOS Syndrome; CHARGE Syndrome; CHIME Syndrome; Christianson-Syndrom; Coffin-Lowry Syndrome; Coffin-Siris Syndrome; Cornelia- de-Lange Syndrome; Donnai Barrow Syndrome; Epilepsia partialis continua; FIRES; Goldenhar Syndrome; Haberland Syndrome; Hermann Pallister Syndrome; Incontinentia pigmenti (Bloch Sulzberger); Jeavons Syndrome; Joubert Syndrome; Kabuki Syndrome; Kleefstra-Syndrome; Kleine-Levin-Syndrome; Kohlschütter-Syndrome; Lafora Syndrome; Leigh Syndrome; Mabry Syndrome; Malan Syndrome; Mandibulofacial Dysostosis with Microcephaly; Marden-Walker Syndrome; Marfan Syndrome; Marshall-Smith Syndrome; MEGDEL Syndrome; Migrating partial epilepsy of infancy; Monozygotic twins; Morbus Krabbe; Mowat- Wilson-Syndrome; Nicolaides-Baraitser-Syndrome; Niemann-Pick Typ C; Pallister-Hall Syndrome; Pallister-Kilian Syndrome; PEHO Syndrome; Pelizaeus Merzbacher; PHACE Syndrome; Phelan Mc Dermid Syndrome; Pontocerebellar hypoplasia; Prader-Willi Syndrome; PRES; PURA; Schimmelpfennig-Feuerstein-Mims Syndrome; Smith-Kingsmore Syndrome; Sotos Syndrome 2; Stickler syndrome; Tay-Sachs; Twin-Twin Transfusion syndrome, Vanishing Twin syndrome; Unverricht-Lundborg; Walker Warburg Syndrome; Williams-Beuren Syndrome; Wolf-Hirschhorn Syndrome.
**Metabolic Disorders**	Antiquitin deficieny; CDG Type I; Cerebral Folate Deficiency; Cerebrotendinöse Xanthomatose; Glut1 Deficiency; Glycine Encephalopathy / non-ketotic hyperglycinemia; lysinuric protein intolerance; M. Alexander; NCL; Niemann-Pick C; OTC deficiency; PDH deficiency; Pyridoxalphosphat dependent epilepsy; Pyridoxine dependent epilepsy; 5-Hydroxytryptophan deficiency.
**Others**	Agenesia corpus callosum; Bilateral perisylvic polymicrogyria; Medical Cannabis within the NETRE groups; Cobblestone lissencephaly; Double Cortex; Eating Epilepsy; Genetic epilepsy in combination with FCD; Heart failure and pilepsy; Holoprosencephaly; Memantine; Microcephaly with simplified gyral pattern; MOGHE; Myoclonic absences; Polymicrogyria; Quinidine; RANBP2 associated acute necrotizing encephalitis; Reading Epilepsy; Schizencephaly; Septo-Optic Dysplasia; Tonic Upgaze:

**Table 2:** Papers published in which coordinators provided NETRE patients.

**Year of publication**	**First author**	**Title**
2007	Kluger G	Long-term use of Zonisamid in refractory childhood-onset epilepsy (6)
	Kluger G	Role of Rufinamide in the management of lennox gastaut syndrome (childhood epileptic encephalopathy) (7)
	van Baalen A	FIRES: febrile infection responsive epileptic encephalopathies of school age (8)
2008	van Baalen A	Febrile Infection-Related Epilepsy Syndrome (FIRES): a non-encephalitic encephalopathy in childhood (5)
2010	Tro-Baumann B	A retrospective study of the relation between vaccination and occurrence of seizures in Dravet syndrom (9)
2011	Müller A	Low long-term efficacy and tolerability of add-on Rufinamide in patients with Dravet syndrome (10)
	Kramer U	Febrile Infection-Related Epilepsy Syndrome (FIRES): does duration of anesthesia affects the outcome? (11)
	Kramer U	Febrile infection-related epilepsy syndrome (FIRES): pathogenesis, treatment, and outcome: a multicenter study on 77 children (12)
	Vendrame M.	Treatment of malignant migrating partial epilepsy of infancy with Rufinamide. Report of five cases (13)
	Kortüm F	The core FOXG1 syndrome phenotype consists of postnatal microcephaly, severe mental retardation, absent language, dyskinesia and corpus callosum hypogenesis (14)
	van Baalen A	Febrile infection-related epilepsy syndrome without detectable autoantibodies and response to immunotherapy: a case series and discussion of epileptogenesis in FIRES (15)
2012	Appenzeller S	Febrile Infection-Related Epilepsy Syndrome (FIRES) is not caused by SCN1A, POLG, PCDH19 mutations or rare copy number variations (16)
	Lotte J	Bromide in patients with SCN1A-mutations manifesting as Dravet syndrome (17)
	von Stülpnagel	First long-term experience with the orphan drug rufinamid in children with myoclonic-astatic epilepsy (Doose syndrome) (18)
	Lemke	Mutations in GRIN2A cause idiopathic focal epilepsy with rolandic spikes (19)
2013	Suls A	*De novo* loss-of-function mutations in CHD2 cause a fever-sensitive myoclonic epileptic encephalopathy sharing features with Dravet syndrome (20)
	Kluger G	Generalized epilepsy in two patients with 5p duplication (21)
	Lal D	DEPDC5 mutations in genetic focal epilepsies of childhood (22)
2014	Barba C	Co-occurring malformations of cortical development and SCN1A mutations (23)
	Ramantani G	Epilepsy in Aicardi-Goutières Syndrome (24)
	Larsen J	The phenotypic spectrum of SCN8A encephalopathy (25)
2015	Lange M	47 patients with FLNA associated periventricular nodular heterotopia (26)
	Nicita F	Epilepsy is a possible feature in Williams-Beuren syndrome patients harboring typical deletions of the 7q11.23 critical region (27)
	Biro A	Effectiveness and tolerability of perampanel in children and adolescents with refractory epilepsies. First experiences (28)
	von Stülpnagel C	SYNGAP1 mutation in focal and generalized epilepsy: an overview and a case report with special aspects of the EEG (29)
	Stamberger H	STXBP1 encephalopathy: a neurodevelopmental disorder including epilepsy (30)
2016	van Baalen A	Febrile infection-related epilepsy syndrom: clinical review and hypotheses of epileptogenesis (31)
	Mignot C	Genetic and neurodevelopmental spectrum of SYNGAP1-associated intellectual disability and epilepsy (32)
	Lotte J	Effectiveness of antiepileptic therapy in patients with PCDH19 mutations (33)
	Müller A	Retrospective evaluation of low long-term efficacy of antiepileptic drugs and ketogenic diet in 39 patients with CDKL5-related epilepsy (34)
	Li D	GRIN2D recurrent *de novo* dominant mutation causes a severe epileptic encephalopathy treatable with NMDA receptor chanel blockers (35)
	Wolff M	Genetic and phenotypic heterogenity suggest therapeutic implications in SCN2A-related disorders (36)
2017	von Stülpnagel C	Epilepsy in patients with GRIN2A alterations: genetics, neurodevelopment, epileptic phenotype, and response to anticonvulsive drug (37)
	Möller R	Mutations in GABRB3: from febrile seizures to epileptic encephalopathies (38)
	Mitter D	FOXG1 syndrome: genotype-phenotype association in 83 patients with FOXG1 (39)
	Depienne C	Genetic and phenotypic dissection of 1q43q44 microdeletion syndrome and neurodevelopmental phenotypes associated with mutations in ZBTB18 and HNRNPU (40)
	Zagaglia S	Neurological phenotypes associated with COL4A1/2 mutations: expanding the spectrum of disease (41)
2018	von Stülpnagel C	Chewing induced reflex seizures (“eating epilepsy”) and eye closure sensitivity as a common feature in pediatric patients with SYNGAP1 mutations: review of literature and report of 8 cases (42)
	Lotte J	Seizure freedom in patients with Dravet syndrome with contraceptives: a case report with two patients (43)
	Wolking S	Clinical spectrum of STX1B-related epileptic disorders (44)
2019	Hofmeister B	Co-occurence of two different genetic diseases: a case of valproic acid hepatotoxicity in nicolaides baraitser syndrome (SMARCA2 mutation)- due to a POLG1-related effect? (45)
	Pringsheim M	Structural brain anomalies in patients with FOXG1 syndrome and in Foxg1+/-mice (46)
	Kluger G	Positive short-term effect of low-dose rosuvastatin in a patient with SYNGAP1 associated epilepsy (47)
	Helbig I	Whole-Exom and HLA sequencing in febrile infection-related epilepsy syndrome (48)
2020	Belohlavkova A	Clinical features and blood iron metabolism markers in children with beta-propeller protein associated neurodegeneration (49)
	Pelletier F	Endocrine and growth abnormalities in 4H leukodystrophy caused by variants in POLR3A, POLR3B, and POLR1C (50)

The following is a brief overview of some of the resulting works of the last 15 years.

### FIRES

At the 2005 annual meeting of the German Neuropediatric Society, three patients with refractory status epilepticus after an unspecific infection were first reported by GK (4). In the search for further therapeutic options, he reached out to his network of national and international colleagues. This was the birth of NETRE. In collaboration with author AvB and colleagues, they coined the term FIRES and described it further. FIRES itself is a very rare epilepsy syndrome with an estimated annual incidence of 1 in 1,000,000 children. It is defined by three phases: Initially, a simple febrile infection occurs in previously healthy children, followed a few days later by recurrent seizures or a highly refractory status epilepticus, often without fever, which leads to a chronic phase with refractory epilepsy and neuropsychological impairment. In a multicenter retrospective case series, 22 previously healthy children were initially described. All of them showed the classic three-phase course and had no evidence of encephalitis, as the cerebrospinal fluid did not show any infection, nor did brain imaging or brain biopsies. Therefore, they proposed the new term “febrile infection-related epilepsy syndrome” (FIRES) (5).

In search of the pathological mechanism, the sera of 12 children were examined, to test the hypothesis that antineuronal antibodies caused FIRES. However, none of the autoantibody tests were positive, in any of the patients. These results, together with the ineffectiveness of first-line immunotherapy in FIRES, cast doubt on the role of an autoimmune encephalitis in the epileptogenesis of classical FIRES (15). Since FIRES patients, together with Dravet patients and *PCDH19* patients, show some common clinical phenotypes, the group around Andreas van Baalen further investigated the three candidate genes *SCN1A, PCDH19*, and *POLG* in 15 patients with FIRES, and looked for copy number variants as risk markers for epilepsy. Since they could not find any pathogenic mutation in these genes, they concluded that these genes are not responsible for FIRES (16). Whole exome sequencing in 50 patients with FIRES revealed no pathogenic variants in any of the established genes for epilepsies. Additionally, HLA sequencing in 29 patients with FIRES failed to identify prominent HLA alleles. Therefore, the underlying etiology of FIRES remains elusive, requiring novel approaches to identify the underlying factors (48).

### SCN2A

Mutations in the *SCN2A* gene, which encodes a voltage-dependent sodium channel, are associated with a peculiar spectrum of neurodevelopmental and epileptic disorders, including neonatal-onset epileptic encephalopathies (e.g., Ohtahara syndrome, epilepsy with migrating focal seizures), infantile epilepsies (e.g., West syndrome), later onset epilepsies, as well as intellectual disabilities with autism. Wolff et al. (36) were able to describe 71 novel cases with *SCN2A*-related disorders. This work was significantly promoted by NETRE, from which the first patients were collected. It is to date the largest published cohort of *SCN2A* patients. In addition to a precise delineation of the different phenotypes, it was shown that age at seizure onset and phenotype correlate with the underlying function of the sodium channel and treatment effects: In children with early infantile epilepsy onset (<3 months of age), mutations resulted in a gain-of-function effect on the sodium channel, whereas later-manifesting forms were associated with a loss-of-function effect. Accordingly, a striking correlation with the effect of sodium channel blockers (SCB) was demonstrated, with relevant implications for treatment decisions (36).

Episodic ataxia is another phenotype in *SCN2A-* related disorders. Schwarz et al. (51) described a cohort of 21 patients. Most of them presented with seizures beginning within the first 3 months of life. Onset of episodic ataxia ranged from 10 months to 14 years of age. Cognitive outcomes were favorable in most patients. Regarding treatment with acetazolamide, data showed a conflicting response in eight patients: Three patients profited from acetazolamide treatment, while the others did not.

### PCDH19

Besides *SCN1A*, the *PCDH19* (protocadherin 19) gene is another gene that leads to epileptic seizures between the 6 and 36th month of life, can also be triggered by fever especially in the beginning, and which occurs in clusters, so that the clinical picture is very similar to Dravet syndrome. It is located on chromosome Xq22.1, and causes the clinical picture of infantile epilepsy, with intellectual disability in girls (52). In a retrospective multicenter study within NETRE, Lotte and colleagues investigated the response to various antiepileptic drugs in 58 *PCDH19* patients. The most effective antiepileptic drugs in this patient group were bromide and clobazam, both after 3 months and after 1 year of treatment. These drugs showed a response rate of 68 and 67%, defined as a 50% reduction in seizures. Sodium channel blockers were less likely to worsen epilepsy in these patients than in Dravet patients and were even effective in some *PCDH19* patients (33).

### CDKL5

Another gene that predominantly affects girls is *CDKL5* (Cyclin-Dependent-Kinase-Like 5). This gene was first described by Montini et al. It is located on chromosome Xp22.13 distal to the ARX gene, which explains the high prevalence in females, however, cases in males with this disease pattern have also been described, but often with a more serious affection/ clinical course. In the first 6 months of life, epileptic seizures that are difficult to treat occur, and the children show pronounced muscular hypotension. Further clinical signs are hand stereotypes and pronounced psychomotor developmental delay with an unremarkable history. This clinical picture is therefore often referred to as the *CDKL5* variant of Rett syndrome. In contrast to Rett syndrome, epilepsy manifests itself much earlier, and the early childhood development is conspicuous even before the onset of epilepsy (53).

Before Müller et al. started their survey on the response to different anticonvulsants in *CDKL5* patients, data on treatment options were only available very sporadically for this patient collective (34). In 2008, Bahi-Buisson observed a positive effect of vigabatrin (*n* = 3), valproic acid (*n* = 2) and in one patient, topiramate, in their cohort of 6/20 *CDKL5* patients with partially controlled seizures (54). Positive effects of a ketogenic diet, vigabatrin and topiramate were also reported in six children in another study (55). NETRE was, therefore, able to record data for a total of 39 *CDKL5* patients internationally, by means of a multicenter study. In this study group, the response to the ketogenic diet and different anticonvulsants was examined after 3, 6, and 12 months. It was shown that after 3 months 27/39 patients (69%), and after 12 months 9/38 patients (24%) showed a 50% reduction of seizures through treatment with anticonvulsant or a ketogenic diet. The most effective drugs were felbamate in 3/3 patients, clobazam in 4/17 patients, lamotrigine in 5/23 patients and zonisamide in 2/11 patients, as well as the anticonvulsant vigabatrin in 8/25 patients and valproic acid in 7/34 patients. An important observation was that many patients showed only a temporary response to drugs (34).

### FOXG1

*FOXG1*-syndrome was considered the “congenital variant of Rett syndrome” when it was first described with a severe neurodevelopmental impairment and its typical clinical features microcephaly (congenital or secondary), dyskinetic and stereotypic movement disorder, absent speech development, feeding problems, gastroesophageal reflux, abnormal sleeping patterns, and early start of therapy refractory epilepsy. The *FOXG1* gene is located on chromosome 14q12 and belongs to the forkhead family, encoding a transcription factor, which is responsible for the development of the ventral telencephalon. Duplications, deletions, frameshifts and point mutations are described as being responsible for characteristic phenotypes. The first report of a patient with a *FOXG1* mutation was published in 2005 (46).

The NETRE working group began with five patients with a *FOXG1* mutation, and the symptom attracting attention among colleagues most was gelastic seizures in four of the five patients, without detection of a hypothalamic hamartoma. However, neuroimaging showed other characteristic changes: Corpus callosum abnormalities, a simplified gyral pattern, reduced white matter volume in the frontal lobes, and frontal pachygyria in a few cases. These results were presented in poster form in 2014 at the annual national neuropediatric meeting in Germany. Together with another group investigating rare neurologic diseases in childhood, *ESNEK* (Erhebung seltener neurologischer Erkrankungen im Kindesalter), Mitter et al. collected data of 83 *FOXG1* patients 2 years later, focusing on the analysis of the genotype-phenotype correlation, and revealed a greater variability of the phenotype than previously described (39).

In a second study, 34 MRI findings of the previously described patients with *FOXG1* mutation were reanalyzed by Pringsheim et al., leading to the identification of characteristic brain anomalies, corpus callosum anomaly in 82%, thickening of the fornix in 74%, and a simplified gyration in 56%. The authors concluded that this triad in the MRI is highly characteristic for the *FOXG1* syndrome and may help in the diagnosis (46).

### GRIN2A

*GRIN2A*, a gene very rarely identified during infancy, as it is more likely to cause epileptic syndromes from infancy onwards, shows a variety of idiopathic focal epilepsies ranging from Rolandic epilepsy to the more difficult to treat courses such as Landau-Kleffner syndrome and Continous Spike Wave During Slow Wave Sleep Syndrome (CSWS). Both Lemke et al. and Lesca et al. were able to show that in individual cases *GRIN2A* mutations are found in these epilepsies, especially in the more severe courses (19, 56).

Therefore, we were interested in whether patients with *GRIN2A* mutations benefit from the anticonvulsants used in idiopathic focal epilepsy. With NETRE, 19 patients with *GRIN2A* mutations could be recruited, seven of which were diagnosed as pathogenic or likely pathogenic according to ACMG criteria. The patients' very heterogeneous seizures responded positively to valproic acid, sultiame, and steroids. Valproic acid improved seizures in four out of five patients, sultiame and steroids in three out of five patients, respectively. However, a final treatment recommendation cannot yet be derived from this small patient group (37).

### COL4A1/2

The autosomal dominant inherited gene mutations of the collagen type IV genes *COL4A1* and *COL4A2* lead to very heterogeneous disease patterns from child- to adulthood. Clinically, they can present as difficult-to-treat epilepsies with microcephaly, as Walker-Warburg syndrome, as familial porencephaly, or as hereditary angiopathy with nephropathy, aneurysms and muscle spasms (HANAC syndrome). Since collagen IV is an essential component of the basal membrane, many of the clinical symptoms can be explained as the consequence of intracerebral hemorrhages. For example, cranial imaging in these patients often reveals porencephalic cysts, periventricular leukomalacia, or lacunar infarctions, as well as gyration disorders in the sense of polymicrogyria. Eyes or kidneys may also be affected in these patients (57).

In the work of Zagaglia et al., 44 newly diagnosed and 55 previously described *COL4A1* and *COL4A2* patients were analyzed concerning neurological phenotypes. Therapy refractory focal epilepsy with an onset in childhood and status epilepticus was the most frequent phenotype in this study group. In 46.4% of the newly diagnosed patients, cranial MRI anomalies were consistent with focal discharges in the EEG. The evaluation of 15 pedigrees leads to the suspicion that in the following generations, the clinical symptoms will be more severe, especially if the gene mutation is inherited from the mother (41).

Nine patients with *COL4A1/-2* mutations and preoperative monitoring were identified in the Center for Pediatric Neurology, Neurorehabilitation and Epileptology, Schoen Klinik, Vogtareuth, Germany. In all patients, neuroimaging showed periventricular leukomalacia and ventriculomegaly suggesting intrauterine cerebral hemorrhage. Moreover, cortical malformations of various degrees were found in all patients of this cohort.

Three patients underwent epilepsy surgery. In two patients, a hemispherotomy was carried out, and one patient underwent a multilobar resection/disconnection. Histological findings in patient 1, who carries a *COL4A1* mutation, showed a focal cortical dysplasia IIID and hippocampal sclerosis. In patients 2 and 3, carriers of the *COL4A2* mutation, a mild malformation of cortical development (MCD) and gliotic changes were found.

Based on the findings in this cohort and of the literature review, two main mechanisms should be considered as leading to neurologic phenotypes, and particularly to epileptogenesis in *COL4A1/2* patients: On one side, CNS impairment is the result of vascular insults, as well as of disseminated microbleeds, which are caused by vascular fragility due to basement membrane impairment in *COL4A1/-2* mutations. On the other side, malformations of cortical development, which can result from impaired function of the pial basement membrane during neuronal migration and cortical organization, contribute to diffuse functional CNS impairment and epileptogenesis in *COL4A1/-2* mutations (58, 59).

Importantly, timing and localization of injury influence the clinical course in *COL4A1/-2* associated epilepsy. If the CNS injury and epilepsy show a focal character, a surgical approach can be discussed. The success of epilepsy surgery is heterogeneous, depending on variable phenotypes in this condition. In most cases, the surgical approach is non-curative and has a low chance of leading to a seizure-free outcome. However, epilepsy surgery can reduce the severity of seizures or stop the encephalopathic course of epilepsy and may enable neurocognitive improvement.

### Nicolaides-Baraitser Syndrome

*SMARCA2* mutations (which codes for the probable global transcription activator SNF2L2) cause Nicolaides-Baraitser syndrome (NCBRS), which is associated with intellectual disability, congenital malformations, particularly of the face and limbs, and epilepsy that is often difficult to treat. With the help of the NETRE and NCBRS support groups, 25 NCBRS patients were recruited. They were retrospectively evaluated for their epilepsy, EEG and MRI findings. The seizures in NCBRS patients were mainly generalized. For anticonvulsive therapy, there was usually only an initial positive response to anticonvulsive therapy, but very rarely a complete absence of seizures. MRI findings in this cohort were unremarkable despite dysmorphic features, so the authors concluded that the etiology of epilepsy is non- structural in this syndrome (58). It is also interesting to note the so-called “double problem,” the simultaneous occurrence of two rare diseases in one patient. In addition to *SMARCA* mutations, one NCBRS patient also had a *POLG1* mutation which, when treated with valproic acid, led to liver hepatotoxicity (59). Although these diseases are rare, the risk of simultaneous occurrence should not be underestimated, and is particularly high in consanguine families (60). According to the figures reported so far from the larger studies, the rate for two or more genetic findings is between 1.4 and 7.2% (average 4.3%) (45).

### Williams-Beuren Syndrome

NETRE also investigates structural chromosomal anomalies. One example of microdeletion syndromes is Williams-Beuren syndrome. This syndrome is caused by a microdeletion on chromosome 7. Phenotypically, these children show varying degrees of cognitive impairment, nutritional disorders in infancy, microcephaly, and dwarfism. Cardiovascular and renal abnormalities are common. Since epileptic seizures in Williams-Beuren syndrome are rarely reported and preliminary studies on the EEG findings in these patients are lacking, Nicita et al. conducted a study in nine patients to investigate whether major atypical deletions are more likely to lead to a neurological phenotype. They found that epilepsy could occur in both groups of Willims-Beuren syndrome, in those with typical, as well as in those with atypical deletions (61).

### SYNGAP1

One of the most extensively researched genes within NETRE is *SYNGAP1* (Synaptic Ras GTPase-activating Protein 1). In 2015, one patient presented with idiopathic epilepsy and intellectual impairment. By this clinical picture, a *SYNGAP1* mutation was found etiologically. New was the finding of EEG normalization through opening of the eyes in a *SYNGAP1* patient (62). The photosensitivity and the good response to valproic acid were confirmed in a following larger study, in cooperation with international colleagues in 17 additional *SYNGAP1* patients. Patients revealed an increasing intellectual impairment over time, in eight patients also autism, as well as for neurologically symptoms ataxia and muscular hypotension. Sixteen out of 17 patients had epilepsy, most of them showed myoclonic-astatic seizures and eyelid myoclonus. Seizure triggers were frequent (seven out of 17 patients) and in one patient, seizures were triggered by chewing (27). With the further registration of *SYNGAP1* patients in the NETRE database, and with the support of the parent support group in Germany, it became clear that seizures triggered by chewing or oral stimulation are a pathognomonic sign of *SYNGAP1*. This was well-documented in a total of eight *SYNGAP1* patients using EEG and video recordings of eating (29). A parallel Australian study by Vlaskamp et al. also reported eating seizures in *SYNGAP1* patients (32).

Since the Ras-Raf-MEK-ERK signaling pathway is negatively regulated by *SYNGAP1*, it is hypothesized that the *SYNGAP1* loss of function mutation leads to less protein being available. This is thought to cause a higher regulation of this pathway, leading to increased excitatory synapse transmission, which may explain the epileptogenicity of *SYNGAP1* (42). In addition to the known cholesterol-lowering effect, statins show further positive effects, mainly due to the inhibition of the intermediates of the mevalonate pathway, which leads to the downregulation of the Ras-Raf-MEK-ERK signaling pathway. This approach led to an individual healing trial with rosuvastatin in our index patients described in 2015. Rosuvastatin was well-tolerated and led to a significant improvement in seizures, even throughout the long-term follow-up of 18 months (63). Encouraged by this positive experience, a pilot study with five additional *SYNGAP1* patients is currently being conducted.

### SYN1

The *SYN1* gene encodes synapsin, which belongs to the family of neuronal phosphoproteins, and is associated with membranes of small vesicles. It is involved in synaptic neurotransmission, plays a role in synaptogenesis, as well as in neuronal development, and the maintenance of synaptic plasticity (64). Mutations in the *SYN1* gene can follow an X-linked recessive, but also an X-linked dominant inheritance. Mutations in the *SYN1* gene are associated with focal epilepsy, a variety of neurological developmental disorders, such as learning disabilities, behavioral disorders, cognitive impairments, and autism spectrum disorders (ASD). Of particular interest is that the *SYN1* gene causes specific reflex epilepsy, namely bathing or water-induced epilepsy (65, 66). Reflex epilepsy refers to recurrent epilepsies that are triggered by very specific stimulation (motor, sensory, or cognitive) and lead to cortical or subcortical hyperexcitability. This genetic hyperexcitability of neuronal structures associated with an *SYN1* mutation leads to water-induced epilepsy. Although hot-water epilepsy and bathing epilepsy are both reflex epilepsies associated with water hyperexcitability, and are therefore often used synonymously or confused, they are very different in terms of genetic background, triggering, appearance, and associated comorbidities.

Bathing epilepsy seems to be triggered by bathing, showering, brushing teeth or washing hair, but the seizure does not seem to occur with the direct stimulus (e.g., bathing) immediately, but only after bathing. The idea of bathing or waiting for a bath can also lead to seizures. To date, only very few cases have been described in the literature, with this appearance and the *SYN1* mutation (47, 67–70). Preliminary analysis of clinical, electro-clinical, and genetic characterization of a larger number of patients with bathing epilepsy and *SYN1* mutations is underway (Accogli et al., in preparation). Further cooperation is currently being established with the genetic group of Christel Depienne (Institute for Human Genetics, Essen) to study the mutation spectrum and mechanisms of this interesting form of reflex epilepsy.

### Early Neuroimpaired Twin Entity (ENITE) and Epilepsy

In the last 30 years, GK has followed more than 400 twin patients in his neuropediatric practice. Within NETRE he is especially interested in twins with genetic forms of epilepsy, as well as in patients with epilepsy following “vanishing-twin-syndrome “(VTS) or ‘'twin-twin-transfusion syndrome” (TTTS). Specifically, the rate of neuropediatric patients with a history of twins (both monozygotic and dizygotic) in the first trimester of pregnancy seen by him has increased within the last 10 years. To test this intriguing hypothesis, this phenomenon has been coined the “Early Neuroimpaired Twin Entity” (ENITE) (see Table 3).

**Table 3:** Description of early neuroimpaired twin entity (ENITE).

**Fertilization**	**Normal or ART (increasingly frequent)**
1st trimester of pregnancy	Complicated cleavage, nidation and embryogenesis due to doublet/multiple pregnancies, VTS in individual cases
2nd and 3rd trimester of pregnancy	Intrauterine cerebral hemorrhage and/or TTTS can occur
Birth	Normal or premature birth or perinatal asphyxia
Variable clinical presentation of ENITE	Dysmorphic features (such as microcephaly, club foot) congenital heart defects cortical malformations (such as lissencephaly, FCD type IIIc/d, MCD) hydrocephalus cerebral palsy mental retardation epilepsy
Epilepsy as optional feature in ENITE	i.a. atypical benign partial epilepsy, structural epilepsy due to cortical malformations. In patients with genetic epilepsies, ENITE can influence the phenotype (as “second hit” or “epigenetic factor”)

*ART, assisted reproduction technology; VTS, vanishing-twin syndrome; TTTS, twin-twin-transfusion syndrome; FCD, focal cortical dysplasia; MCD, mild malformation of cortical development*.

This observation may be biased through the longstanding interest in the use of assisted reproduction technology (ART) for becoming pregnant. In Germany, the number of births from ART has increased significantly in the last 15 years, which has led to a higher number of twin pregnancies (71). Both ART (72, 73) and twin pregnancies, and the complications associated with them (e.g., VTS, TTTS, intracerebral hemorrhage, fetal malformations, premature delivery, and asphyxia), have an increased risk of neurological disease and epilepsy (74–76).

In NETRE we follow five pairs of monozygotic twins (1 *SCN1A*, 1 *RNASEH2B*, 3 *STXBP1*), This creates a special opportunity to assess pregnancy and birth factors as important explanatory factors for variable phenotypic expressions in genetic epilepsies. For *RNAESH2B*, one twin has the atypical presentation of Aicardi Goutières syndrome, while her monozygotic twin sister is healthy (at age 4 years). Both twins with *SCN1A* mutation show a very similar presentation of Dravet syndrome. Among the three pairs of monozygotic twins with *STXBP1* mutations, two pairs have a very similar and typical phenotype of an *STXBP1* mutation. However, in one pair the overall clinical presentation is divergent among the two twin boys, including heart failure in only one boy. This difference in the clinical manifestation of a genetic form of epilepsy in monozygotic twins with *de novo* mutations might at least in part be explained by a putative variable level of postzygotic somatic mosaicism in the critical cerebral cortex between twins. Other genetic factors, like variable imprinting effects, might be discussed also, but are currently not established (77). We propose further investigation of the “ENITE” cohort, by recruiting discordant monozygotic twins with epilepsy, and to thereby learn more about these cases. Accordingly, Brodtkorb et al. (78) suggested that monochorionic twinning might be a risk factor for regional defects in neuronal migration, presenting for example as focal cortical dysgenesis as FCD type IIIc/d. Whether or not twinning might also increase the risk of other, previously established monogenic forms of defective neuronal migration remains to be assessed. Further epidemiological studies including prospective controlled studies in epilepsy patients born after normal fertilization or ART, and studies in larger cohorts of patients with clear VTS and/or TTTS syndrome are essential to further unravel genetic or non-genetic cofactors contributing to phenotypic variability in genetic epilepsies. We suggest to routinely include in the patient history of a child with epilepsy questioning of the parents about the use of ART or any abnormalities in the first trimester of the pregnancy. Overall, the authors hope that this article will arouse the interest of the scientific community in identifying and describing epilepsy in twins, who were conceived spontaneously or by ART, and who may have vanished unrecognized during early pregnancy.

### Outlook-PATRE

Our new project PATRE (**PAT**ient based phenotyping and evaluation of therapy for **R**are **E**pilepsies) will complement NETRE by gathering information about therapies directly from patients and their caregivers.

There are already many partnerships and interactions of NETRE with parents' self-support groups. Nowadays, the parents in these self-support groups are extremely well-networked worldwide through social media. This makes it easy to access the patients and their parents as a source for data for research on rare pediatric-onset epilepsies. Hence, in our planned project PATRE it is not the treating physician who serves as a partner, but the patients and their parents.

Exemplary, of the already-existent close cooperation between our medical staff and the self-support groups in this field, is the primary description of chewing reflex seizures as a typical feature of SYNGAP1 patients. The first observations were made during an inpatient stay at the Schön Klinik Vogtareuth. A questionnaire sent to members of the self-support group via social media confirmed the suspicion that there is a frequent occurrence of this symptom in SYNGAP1 patients. This enabled timely and targeted clinical observations to follow, which ultimately led to the publication of the initial description of this phenomenon (42).

To ensure a safe and user-friendly method of data collection, the use of modern technology is necessary. Therefore, the project PATRE aims to develop a standardized procedure for researching the phenotype and therapies for rare pediatric epilepsies using electronic parent questionnaires. This creates a basis for many further PATRE projects in the future.

These electronic parent questionnaires will be implemented on a server platform on the internet. This server will meet all data protection and ethical requirements for medical and technical data collection. The server offers an extremely user-friendly interface, making it easy to use, not only for medical staff, but also for parents and patients. Filling out the questionnaires is possible without high technical barriers, even directly on user's smartphones. This project cannot replace direct clinical research in our institution and other centers. Nonetheless, we can obtain data, which can be verified using clinical data from the institutions and NETRE. This can therefore lay the foundation for clinical research in a broad range of rare epilepsies in children.

## Discussion

In 15 years, the benefits of NETRE have been highly appreciated, which is reflected in the increase of daily communication within the NETRE network. As NETRE allows clinicians to quickly exchange information with colleagues at the international level about therapeutic experiences in rare diseases, this information exchange is reassuring not only for clinicians but also for parents with a child affected by a rare disease.

In the last two decades, a genetic etiology has been identified in over half of all epilepsies, and single gene defects in ion channels or neurotransmitter receptors have been associated with most inherited forms of epilepsy, including some focal and lesional forms, as well as specific epileptic developmental encephalopathies (79, 80). This has led to new insights, not only regarding therapeutic options, but also for describing further the clinical phenotype in these rare epilepsies, such as the chewing-induced seizures in *SYNGAP1* patients, or the bathing epilepsy in *SYN1* patients, or the characteristic MRI findings in *FOXG1* patients. With this additional clinical information, it may in the future be possible to diagnose patients earlier (42, 46).

The large number of different syndromes and seizure types, as well as the highly variable inter-individual response to the therapies, makes management of this condition often challenging. The term precision medicine describes the treatment of patients with therapy targeted to their specific pathophysiology (79). Although examples can be found in several areas of medicine, the role of precision medicine in day-to-day healthcare is relatively limited. Gene therapy is very promising, especially in the context of specific, rare epileptic disorders as viral-vector-mediated gene transfer offers the opportunity to design a rational treatment targeted to specific neuronal populations in epileptogenic foci. Nevertheless, the implementation of advanced therapies should be accompanied by the development of advanced tools allowing clinicians to identify patients suitable for clinical trials or newly approved disease-modifying therapies early on.

In collaboration with other groups and networks, personalized therapeutic approaches are still being sought, such as the individual therapeutic trial of rosuvastatin in *SYNGAP1* patients (47). On the other hand, the limitations of currently available therapies have also become apparent, such as the important observation that many *CDKL5* patients showed only a temporary response to anticonvulsants, and therefore the emphasis on avoiding over-treatment in these patients may also be an optional therapeutic goal (34). For some genes, such as *GRIN2A*, which codes for severe forms of idiopathic focal epilepsy, and *PCDH19*, which has similarities to Dravet syndrome, the therapeutic concepts of these forms of epilepsy may also apply to these genes (33, 37). Moreover, not always does a single gene mutation explain the phenotype, such as in our observations of the so-called “double problem” of the simultaneous occurrence of two rare diseases in one patient, or the observations of our new called entity ENITE.

NETRE has also enabled some coordinators to create one of the largest cohorts of patients with specific gene mutations worldwide, and thus, a good overview of therapeutic options (e.g., FIRES, Nicolaides-Baraitser syndrome). The database of many patients with rare epilepsies within NETRE also enables further investigations in all groups, such as the status of special AED treatment or ketogenic diet, as well as on special clinical aspects, such as for instance the question of seizures in the context of vaccination.

The following limitations and concerns have been raised over the years: As each gene group is led by a medical coordinator who is responsible for the data entry form, there is no standardized questionnaire within NETRE. Another problem is the—often non-automatic—return of the data entry forms, and the decreasing interest of some involved medical coordinators, so that not all gene groups are equally active and some of them, therefore, produce fewer results. Last, since every colleague who provides information on a patient has the right to co-author, the list of authors in publications has become quite long, which sometimes leads to problems in the publication process. Up to now the data collection is only retrospectively and in the future, the question of how to use the data prospectively must be addressed. All in all, we have the impression that the fact of being non-sponsored has contributed to the success of NETRE.

A next step will be the PATRE Project (**PAT**ient based phenotyping and evaluation of therapy for **R**are **E**pilepsies), where information on therapies will be obtained directly from patients and their caregivers. We think that this will be a good amendment to NETRE, as parents often treat their children with substances of which they have heard of positive therapeutic effects, and this variable is not yet recorded. We noticed this after the publication of our case report on the positive effects of rosuvastatin in one of our *SYNGAP1* patients, when we talked to further *SYNGAP1* patients (47).

The experience of the last 15 years has been encouraging. Learning from the experiences of colleagues in treating individual cases led to the expertise of the network, and enabled members to offer their patients more information and treatment options. Ideally, the collected cases would be summarized and with their publication, the data would be accessible to a wider audience, who would be able to benefit of representative numbers. Such collaboration would eventually allow physicians to predict more accurately which treatment and prevention strategies will be most effective for a particular disease, in a specific group of people.

## Disclosure

PATRE has the following founding number at the PMU: E-20/31/161-KEK.

## Author Contributions

CS and GK planned the study and took the lead in writing the manuscript. All authors discussed the results, provided critical feedback, and contributed to the final form of the manuscript.

## Conflict of Interest

GW obtained honoraria for speaking engagements from Desitin (Hamburg, Germany) and Novartis (Nürnberg, Germany). He gave scientific advice for PTC Therapeutics (Frankfurt, Germany). PS developed this work within the framework of the DINOGMI Department of Excellence of MIUR 2018-2022 (legge 232 del 2016), has received speaker fees and participated at advisory boards for BioMarin, PTC Therapeutics, Zogenyx, GW Pharmaceuticals, and has received research funding from ENECTA BV, PTC Therapeutics, GW Pharmaceuticals, Kolfarma Srl., Eisai. GK obtained speaker honorary from Desitin (Hamburg, Germany) and Eisai (Frankfurt, Germany). The remaining authors declare that the research was conducted in the absence of any commercial or financial relationships that could be construed as a potential conflict of interest.

## Supplementary Material

Supplementary Data Sheet 1 Example of a data sheet to collect patient's data within NETRE (here for SYNGAP1).
