# Supplementary material for: Network for Therapy in Rare Epilepsies (NETRE): Lessons From the Past 15 Years
Source: Front Neurol. 2021 Jan 14;11:622510. doi: 10.3389/fneur.2020.622510 (PMC7840830; doi:10.3389/fneur.2020.622510)
Supplement: Supplementary Data Sheet 1 — Example of a data sheet to collect patient's data within NETRE (here for SYNGAP1). [file Data_Sheet_1.PDF]

**Patients initials / Dr**
**Published?**
Age at the time of the study (years)
Sex
Ancestry
Family history
Parental age at birth: mother (Mo) father (Fa) in years
**Genetics**
Mutation type
Mutation
Protein level
Location in gene (exon)
Inheritance
Other significant associated genetics abnormalities (CNV, point mutation…)
Method of molecular diagnosis (WES, targeted seq, panel, etc.)
**Pregnancy & neonatal period**
Pregnancy and delivery
Birth length in cm (perc) / weight in g (perc) / head circumference in cm (perc)
Neonatal findings
**Developmental stages**
Age of sitting / walking (m:months)
Age of first words (m:months, y:years) / first sentences
Current language ability
Regressive episode during the development / Age (m:months, y:years)
**Intellectual disability (ID)**
Estimated level of ID
Age at evaluation (y)
Evaluation Scale/mode of evaluaiton
Mean IQ
**Autism spectrum disorder (ASD) if possible ADI / CARS or ADOS scale (indictae here)**
Alteration of nonverbal communication (none / mild / moderate / severe)
Repetitive behaviours (none / mild / moderate / severe)
Stereotypies (none / mild / moderate / severe)
Alteration of social interactions (none / mild / moderate / severe)
Behaviour troubles (describe)
Other
**sleep disorders**
sleep problems yes/no
time until falling asleep (min)
hours awake during night
how often/week
**EPILEPSY**
Age at seizure onset (m:months or y:years)

Seizure type at onset
Seizure types during disease course
Seizures provoked by oral stimulation /eating/head stimulation
other triggers for seizures
Febrile seizures
Status epilepticus (yes/no)
Max frequency of seizures/week
Present frequency of seizures/week
Lifetime / current anti-epileptic treatment
Pharmoresistance (yes if needs several AED or several AED ineffective)
**Electroencephalogram**
Age at examination (years)
Main abnormalities: epileptic features, background activity
photosensitivity tested?
Triggers of seizures
**Other significant comorbidities**
**Clinical Examination**
Age at examination (years)
Height in cm (SD) / weight in kg (SD) / head circumference in cm (SD)
Neurologic examination (describe)
pain insentitivity
ataxia
nystagmus
strabism
Dysmorphic features (describe)
**Brain imaging (MRI)**
age at examination in y:years m:months
main findings
**other tried therapies and effect**
CBD
Statine
other
